# Outcomes of craniotomies for chronic subdural hematoma in Sierra Leone

**DOI:** 10.11604/pamj.2021.38.80.19173

**Published:** 2021-01-25

**Authors:** James Baligeh Walter Russell, M'Baimba Lamin Baryoh, Victor Conteh, Len Gordon-Harris, Durodami Radcliffe Lisk

**Affiliations:** 1 Department of Internal Medicine, Faculty of Clinical Sciences, College of Medicine and Allied Health Sciences, University of Sierra Leone, Freetown, Sierra Leone,; 2 Department of Internal Medicine, Choithrams Memorial Hospital Freetown, Freetown, Sierra Leone,; 3 Department of Surgery, Faculty of Clinical Sciences, College of Medicine and Allied Health Sciences, University of Sierra Leone, Freetown, Sierra Leone,; 4 Department of Surgery, Choithrams Memorial Hospital, Freetown, Sierra Leone

**Keywords:** Chronic subdural hematoma, flap craniotomy, trauma, general surgeon

## Abstract

**Introduction:**

chronic subdural hematoma (cSDH) is not uncommon in sub-Saharan Africa and has a striking morbidity and mortality if not managed adequately. With the limited number of neurosurgeons in resource poor countries, general surgeons should be trained in the skills of craniotomy and burr-hole craniostomy.

**Methods:**

we conducted a retrospective review of all medical records of patients with cSDH, who underwent flap craniotomy at the Choithrams Memorial Hospital, Sierra Leone, between January 2016 and March 2018. The case notes, operative records and computerized axial tomography (CT) scans were reviewed and all pertinent data extracted. All patients were jointly managed post operatively by medical (neurological) and surgical teams in an intensive care unit.

**Results:**

a total of 23 patients had surgical drainage of the chronic subdural hematoma. The mean age of the patients was 65.8 years (ranging from 54-78) with a male: female ratio of 3: 2: 1. The main predisposing risk factors were head trauma (60.9%) and antiplatelet medications (21.7%). Hypertension was the most common comorbidity, followed by diabetes mellitus. Ten (62.5%) out of sixteen patients referred for Head CT-scan by the primary physicians, had an initial missed clinical diagnosis until computerized tomography (CT) scan confirmation report of chronic subdural hematoma (cSDH) was obtained. Flap craniotomy under general anesthesia with a subdural drainage left in situ (100%) was done for all patients. Mean duration of Intensive Care Unit (ICU) admission was 10.6 days (range 6-16 days). Twenty-one (91.3%) patients made a full recovery. There was no mortality.

**Conclusion:**

flap craniotomy for cSDH was safely performed by a traumatologist/general surgeon in a developing country where there is no neuro-surgical service. The outcome of the patients was favorable as there was co-management with the surgical and medical team.

## Introduction

Chronic subdural hematoma (cSDH) represents one of the most common types of intracranial hemorrhage, in the neurosurgical department. Globally, surgical drainage of cSDH is regularly performed by neurosurgeons with good results [1]. Chronic subdural hematoma is the collection of liquefied blood degradation underneath the dura matter that may result in brain tissue compression and subsequent neurological sequalae. However, little is reported in the literature on the safety outcome of surgical drainage for cSDH performed by a traumatologist/general surgeon. Studies from Kenya [2] and Australia [3] have reported successful outcomes from general surgeons. As there is no neurosurgical referral center in Sierra Leone, patients suffering from cSDH carry a high mortality without surgical intervention. Hence there are no published data from Sierra Leone on the surgical intervention of cSDH. The reported incidence of cSDH increase greatly with age, ranging from approximately 3.4 per 100,000 in patients younger than 65 years of age, to 8 to 58 per 100,000 in those older than 65 years [4,5]. To deal with this high disease burden, there should be at least one neurosurgeon for every five million people in sub-Saharan Africa [6]. Varieties of surgical techniques have been used in the treatment of cSDH including twist- drill craniostomy and catheter drainage [7], twist-drill and spontaneous hematoma efflux [8], small craniostomy and endoscopic removal [9], subduro-peritoneal shunt as an alternative for infants [10] and elderly patients [11], large craniotomy, hematoma removal and membranectomy [12], or burr-hole craniostomy with or without continuous closed-system drainage [13,14]. Though simple burr-hole evacuation and irrigation of the subdural cavity has become the gold standard with the best clinical outcome [15], many authors have also advocated for large craniotomy and extended craniotomy with membranectomy [16-18]. The results presented from this retrospective study are the clinical outcomes resulting from the management of cSDH in patients whose surgical operation were performed by a traumatologist/general surgeon in Freetown Sierra Leone.

## Methods

A retrospective review of the medical records including operational logbooks of all patients admitted to Choithrams Memorial Hospital, Freetown Sierra Leone over the period of January 2016 to March 2018 with a clinical diagnosis and management for chronic subdural hematomas. On admission, all patients were jointly managed by the Medical and Surgical Departments of the hospital, wherein these patients underwent complete pre-operative and post-operative examination by the consultant neurologist. All patients, whose chart had a discharged diagnosis of cSDH, were retrieved from the records office. These charts and the operative registry logbook system were then reviewed. Data recorded were, the age and sex of the patients; presenting complaints, clinical examinations findings, investigational findings, operative procedure and findings, mortality, length of hospital stay and clinical outcomes. Any associated comorbidity identified in the chart was recorded. Detailed information of potential risk for cSDH such as history of head injury, initial symptoms and date they appear, alcohol abuse, usage of anticoagulant or antiplatelets medications, hematological, liver cirrhosis, epilepsy and chronic renal failure were extracted. A 64 multi-slice computerized tomography (CT) scans of the head was performed on all suspected cSDH patients and radiological diagnosis was confirmed by a Board-Certified Radiologist in the hospital. A post-operative CT scan of the head was done and also reported by the radiologist. Surgery was performed under general anesthesia and flap craniotomy was performed on the thickest part of the hematoma as determined by CT scan. In all cases, subdural blood was evacuated by repeated irrigation with physiological saline and the patient received one closed subdural drainage system. All post-operative cases from the surgical theatre were immediately admitted into the Intensive Care Unit (ICU) for management by the medical team (led by the neurologist) and the surgical team (led by the operating surgeon). Whenever there was significant clinical improvement in the symptoms and signs of the operated patient in the ICU, they were then transferred to the Surgical Ward. All drainage tubes were removed in the ICU before transfer. In our study, the primary and secondary outcomes are mortality and symptomatic improvement respectively. We defined mortality as any deaths that may occur post-operatively within 30 days regardless of whether it was related to the procedure or not. A patient was considered to have improved if their symptoms or neurological symptoms had improved or resolved from the last follow up. The data were collected using a standardized data collection sheet and the results entered into a microsoft excel spreadsheet and summarized using descriptive statistics.

The study was approved by the Sierra Leone Ethics and Scientific Review Committee. Anonymity was maintained by using independent serially coded numbers which were then assigned to the case records. The extracted data was then handled with strict confidentiality.

## Results

Twenty-five charts with the diagnosis of cSDH were retrieved from the Records Office but only 23 consecutive cases had surgical drainage of the cSDH (Table 1). Of the 2 excluded cases, family members refused consent in one case as they were concerned about the old age of the patient whilst the other patient was medically evacuated to a Neurosurgical Centre outside Sierra Leone. Of the twenty-three operated cases, there was a male: female ratio of 3: 2: 1. The mean age of 65.8 years, ranging from 54-78 years was documented. The commonest predisposing risk factors was head trauma (60.9%), followed by antiplatelet medications (21.7%), significant alcohol use (8.7%), whilst 8.7% were unknown. Antiplatelet medications were considered a predisposing risk factor in a patient if there were no other risk factors other than the antiplatelets medication the patient is taken. None of these patients were on anticoagulants. Nevertheless, none of these predisposing factors had any significant influence on the outcome of the surgical operation. The main clinical signs and symptoms at presentation varied, with most of the patients having headache and altered behavior. Examination revealed that most patients had some amount of cognitive decline or limb weakness. Hypertension was the most common comorbidity, followed by diabetes mellitus. Four patients had more than two comorbidities. Sixteen patients (69.6%) in this study were referred by their primary physicians to the Choithrams Memorial Hospital for further management. There was an initial missed clinical diagnosis of 62.5% (10) of the sixteen patients referred by the primary physician as the diagnosis was only established after Head CT-Scan was done.

**Table 1:** etiology, clinical presentation and comorbidities of patients with chronic subdural hematoma (N = 23)

Aetiology	Frequency (N)	Percentage (%)
Head injury	14	60.9
Antiplatelets	5	21.7
Alcohol use	2	8.7
Unknown	2	8.7
**Main signs and symptoms presentation**		
Headache	15	65.2
Disturbance of consciousness	10	43.5
Hemiparesis	8	34.8
Confusion	7	30.4
Seizure	4	17.4
Speech defect	2	8.7
Gait disturbance	2	8.7
**Comorbidities**		
Arterial hypertension	9	39.1
Diabetes mellitus	5	21.7
Alcohol	4	17.3
Cardiac disease	4	17.3

Out of the 10 patients whose clinical diagnosis were initially missed by their primary physicians, 60% were being managed initially for stroke whilst 40% for seizure disorders. The Glasgow Coma Scale recorded in the study is shown in (Table 2). The majority of the patients (56.5%) had a GCS of 15 whilst (43.5%) patients presented with an altered conscious state. Computerized tomography scan evaluation identified the sites of the hematoma: right side in 17 (73.9%) of the patient and left side in 6 (22.1%). None of the patients in our studies had bilateral haematoma. The surgical technique done by the surgeon was “flap craniotomy”, membranectomy and saline irrigation in all patients. All surgeries on these patients were done under general anesthesia with a subdural drain left in situ in all patients. The drainage tube, was left in place for a median of 4 days and mean of 4.8 days (range 3-8 days) with a duration of intensive care unit stay of 10.6 days (median 8 days, range 6-16 days). Antibiotics therapy was commenced on the day of surgery and continued until the drain was removed. One patient had a second bleed after the first surgery necessitating a second surgery with removal of a massive recurrent clotted hematoma. By the seventh day of admission, 18 (78.2%) patients had made full recovery and at the time of discharge full recovery was recorded in 21 (91.3%) patients. The overall outcome in the majority 22 (95.6%) of patients in our study was favorable, with a recurrence rate of 4.3% (1 patient) and mortality rate of 0%.

**Table 2:** Glasgow coma scale at presentation

GCS	Frequency (N)	Percentage (%)
15	13	56.5
11-14	6	26.1
<10	4	17.4

## Discussion

Intracranial hemorrhage due to cSDH was first described by John Wepfer in the 17^th^century and since 1956 it is been managed by neurosurgeons [19]. It is managed by a simple surgical intervention in which the patients experience a favorable outcome. The results from this study demonstrated that cSDH, can be managed with favorable outcome by a traumatologist/general surgeon in a poor resource setting such as Sierra Leone with a good outcome. In our case series review, surgical procedure was performed by an experienced traumatologist/general surgeon whilst post-operative management was done by a combined medical and surgical team in the hospital of a developing country. This retrospective case review of patients with cSDH to our knowledge is the first case series to describe an operative clinical management of cSDH by an experienced traumatologist/general surgeon, followed by a post-operative co-management by a medical team in sub-Saharan Africa. In lower middle-Income countries (LMIC) such as Sierra Leone, general surgeons have been managing surgical conditions outside their comfort zones in order to provide life- saving therapy to the needy patients. In our study, the mean age of the patients was 65.8 years with a male preponderance of 82.6%. The male preponderance may be attributed to (a) the greater exposure of males to injury such as motorcycle and tricycle accidents, (b) fewer females seeking medical advice, and (c) estrogens may have a protective effect on the capillaries [20]. These findings are similar to data from other centers around the world with the majority of the patients being male in the 6th decade of life or older [21-23]. Headache 61.5% was the most common presenting complaint, which is similar to a study in Ghana by Dakurah *et al*. [24] and Kenya by Kanyi *et al*. [2]. This notwithstanding, there are variations in the incidence of headache as reported in different studies, ranging from 14% to 80% [25,26].

Kaste *et al*. [23] in their series reported headache in 72% and limb weakness in 48%, whereas Dronfield´s [27] series reported cognitive disturbances in 100%. Mckissock and Loud (26) had limb weakness as the most common presenting symptom. Head injury (46.2%) and the use of antiplatelets (30.7%) were the major aetiological factors in their study, while there were no patients on anticoagulant therapy. Our study differs from the studies of Mckissock and Loud [28], Fogelholm *et al*. [29] and Richter *et al*. [30] who documented an average of 52% to 75% incidence of trauma in their series. Arterial hypertension (38%) is the most common comorbid diseases reported in our study. This is similar in frequency as reported in other studies [24-31]. However, the prevalence of hypertension as a co-morbid disease is much higher than the prevalence of hypertension in the general populations as reported by other authors [32-34]. Craniotomy as a treatment of cSDH, was the known optimal surgical technique described by Putnam and Cushing in 1925 despite the high surgical mortality of 30% [35]. However, Mohamed [36] argued for craniotomy and outer membranectomy with minimal incidence of recollection, morbidity, and mortality. All patients in this study had flap craniotomy, physiological saline wash and closed drainage. The favorable outcome seen in our study could be attributed to the small sample size of our patients and the co-management by the surgical and medical team. As a private hospital, patients had to pay out-off pocket fees for all hospital medical bills and this could account for the sample size as most patients in Sierra Leone cannot afford private hospital management. The use of drain in these patients was not found to be associated with increased surgical complications [37,38]. The rate of re-operation was 4.3% and this is consistent with other studies which is in the range between 5.5 and 25% [39-42]. Since Choithrams Memorial Hospital is a privately owned institution, cost constraints and the burden to pay hospital bills by patients accounted for the small sample size in our cases series and this could reflect a patient selection bias. Although our sample is small, our data suggests that surgical intervention by an experienced traumatologist/general surgeon and commitment of the supervising physicians post - operatively may be associated with improved patient outcome and survival.

## Conclusion

The results of our study clearly indicate that there was a favorable outcome of flap craniotomy performed by a traumatologist/general surgeon for cSDH in a country where there is no neuro-surgical service. This favorable outcome could be attributed to the continuous monitoring and management by the Medical Team alongside with the surgical team. This study emphasizes the importance of training general surgeons in resource poor countries the skills of performing craniotomy or burr-hole craniostomy, thus patients suffering from the cSDH should be able to access adequate care thereby preventing untimely death.

### What is known about this topic

- Chronic subdural hematoma is the collection of blood between arachnoid membranes and the inner layer of the dura;
- For patients with symptoms and significant mass effect due to cSDH, the main stay of management is by surgical evacuation by burr-hole craniostomy or craniotomy with or without irrigation and drainage;
- This procedure is commonly performed by neurosurgeon.

### What this study adds

- In the absence of neurosurgeon in the poor resources setting, surgical evacuation of cSDH can be performed a traumatologist/general surgeon using a flap craniotomy with irrigation and drainage;
- This study emphasizes the importance of general surgeons in developing countries working outside their comfort zone to provide life-altering therapy to patients in need.
